# A robust SNP barcode for typing *Mycobacterium tuberculosis* complex strains

**DOI:** 10.1038/ncomms5812

**Published:** 2014-09-01

**Authors:** Francesc Coll, Ruth McNerney, José Afonso Guerra-Assunção, Judith R. Glynn, João Perdigão, Miguel Viveiros, Isabel Portugal, Arnab Pain, Nigel Martin, Taane G. Clark

**Affiliations:** 1 Faculty of Infectious and Tropical Diseases, London School of Hygiene and Tropical Medicine, London WC1E 7HT, UK; 2 Faculty of Epidemiology and Population Health, London School of Hygiene and Tropical Medicine, London WC1E 7HT, UK; 3 Centro de Patogénese Molecular, Faculdade de Farmácia da Universidade de Lisboa, 1649-003 Lisboa, Portugal; 4 Grupo de Micobactérias, Unidade de Microbiologia Médica, Instituto de Higiene e Medicina Tropical, Universidade Nova de Lisboa, 1349-008 Lisboa, Portugal; 5 Biological and Environmental Sciences and Engineering (BESE) Division, King Abdullah University of Science and Technology, Thuwal 23955-6900, Kingdom of Saudi Arabia; 6 School of Computer Science and Information Systems, Birkbeck College, London WC1E 7HX, UK

## Abstract

Strain-specific genomic diversity in the *Mycobacterium tuberculosis* complex (MTBC) is an important factor in pathogenesis that may affect virulence, transmissibility, host response and emergence of drug resistance. Several systems have been proposed to classify MTBC strains into distinct lineages and families. Here, we investigate single-nucleotide polymorphisms (SNPs) as robust (stable) markers of genetic variation for phylogenetic analysis. We identify ~92k SNP across a global collection of 1,601 genomes. The SNP-based phylogeny is consistent with the gold-standard regions of difference (RD) classification system. Of the ~7k strain-specific SNPs identified, 62 markers are proposed to discriminate known circulating strains. This SNP-based barcode is the first to cover all main lineages, and classifies a greater number of sublineages than current alternatives. It may be used to classify clinical isolates to evaluate tools to control the disease, including therapeutics and vaccines whose effectiveness may vary by strain type.

## Abstract (web summary)

Genetic variation in *Mycobacterium tuberculosis* complex (MTBC) bacteria is responsible for differences in factors such as virulence and transmissibility. Here, the authors analyse the genomes of 1,601 MTBC isolates from diverse geographic locations and identify 62 SNPs that may be used to resolve lineages and sublineages of these strains.

## Graphical abstract

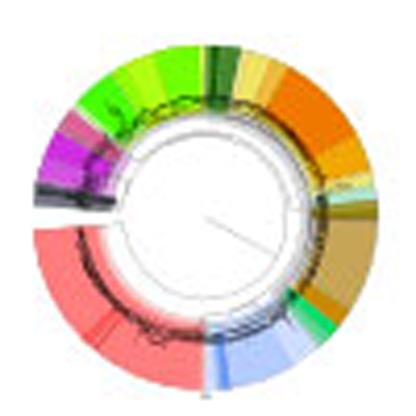

Infection with bacteria of the *Mycobacterium tuberculosis complex* (MTBC) results in a variety of outcomes including latent infection and/or progression to pulmonary or extra-pulmonary manifestations of disease. Such diversity has been historically attributed to host and environmental factors, and the MTBC was previously considered genetically monomorphic in nature^1^. However, the development of typing methods that discriminate strains into distinct lineages has demonstrated previously unrecognized diversity. The first attempts to differentiate strains were based on phage typing^2^. Geographic patterns of strain distribution were identified and in the south of India, an association with virulence was noted^3^. Phage typing has since been superseded by genetic methods. Initial efforts were focussed on large sequence polymorphisms^4^. Six major global MTBC lineages have been defined (1 Indo-Oceanic, 2, East-Asian including Beijing, 3 East-African-Indian, 4 Euro-American, 5 West Africa or *Mycobacterium africanum* I, 6 West Africa or *M. africanum* II), distinct from a *M. bovis* clade. Lineages 1, 5 and 6 are considered ‘ancient’, and 2 to 4 ‘modern’. A novel phylogenetic lineage of MTBC that appears to be intermediate between the ancient and modern has been described in Ethiopia and the Horn of Africa^5^,^6^, referred to as lineage 7. Additional genotyping methods have been developed based on detection of insertion elements, variable number of repeats or the presence or absence of spacer oligonucleotides (spoligotyping)^7^. Although mainly used for public health purposes or epidemiological studies, spoligotype families have been used to provide further resolution within the lineages^8^.

Lineage is of importance to tuberculosis control as it has been shown that strain type may play a role in disease outcome, variation in vaccine efficacy^9^ and emergence of drug resistance^10^. Different strains of MTBC have produced distinct biological responses in experimental models and can affect clinical presentation^11^,^12^,^13^. Strain type may also influence disease epidemiology as in some settings it is associated with the presence or absence of clustering due to recent transmission^14^. Lineage-specific differences in the virulence of clinical isolates have been reported across independent experimental systems with modern lineages, such as Beijing and Euro-American Haarlem strains believed to exhibit more virulent phenotypes compared with ancient lineages, such as East-African-Indian and *M. africanum* strains^15^. The molecular mechanisms and genetic factors responsible for the described differences remain largely unknown. Their investigation requires transparent, easily applied, reliable methods for determining stain type. Several sets of single-nucleotide polymorphisms (SNPs) have previously been proposed^16^,^17^,^18^,^19^,^20^ but limited numbers and variation of strains were used in their construction and they describe a restricted number of lineages and sublineages.

In this study, we have sought to provide an improved system by examining whole-genome data from a large number of strains (*n*=1,601) from a diverse geographic spread. We identify a single panel of 62 SNPs that may be used to resolve all seven lineages and a total of 55 sublineages.

## Results

### Population structure

**Figure 1:**
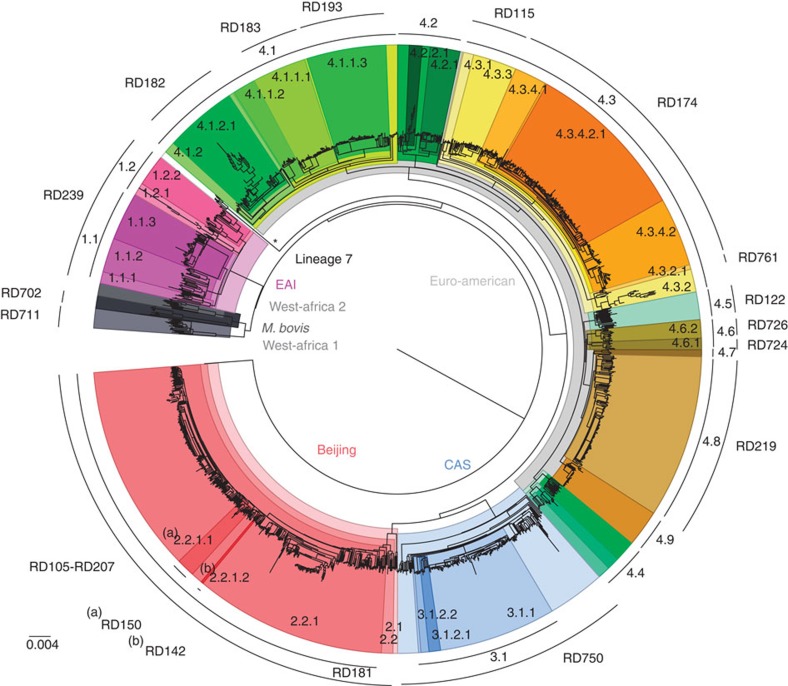
Global phylogeny of 1,601 MTBC isolates. A total of 91,648 SNPs spanning the whole genome were used to reconstruct the phylogeny of 1,601 MTBC isolates. All seven main MTBC lineages are indicated at the inner area of the tree. The main sublineages are annotated at the outer arc along with lineage-specific RDs. Identified clades are colour coded.

A genomic analysis was performed on whole-genome sequences of 1,601 MTBC isolates from eleven independent sequencing studies from different areas of the world, with representation of all seven major lineages (1, *n*=121, 7.6%; 2, *n*=390, 24.3%; 3, *n*=189, 11.8%; 4, *n*=856, 53.5%; 5, *n*=17, 1.1%; 6, *n*=11; 7, *n*=6), as well as *M. bovis* (*n*=11; Supplementary Table 1). A total of 91,648 SNPs were identified; 54.6% were observed in a single sample, that is, not found in other samples (Supplementary Fig. 1), 89.2% were in coding regions, and 63.5% resulted in non-synonymous changes in amino acids. The SNP-based phylogenetic tree demonstrated a clustering largely congruent with published MTBC phylogenies (Fig. 1). MTBC main lineages (1–7 and *M. bovis*) and sublineages were subsequently identified based on the spoligotype and regions of difference (RD) composition of the clades in the SNP-based phylogeny (see Methods). The phylogeny revealed the presence of new clades for which RDs do not discriminate. In particular, there were gaps in the Euro-American lineage for which molecular fingerprint classifications are less accurate^16^.

Although estimates of genetic diversity may be influenced by sampling bias, the greatest nucleotide diversity was observed in lineages 1 (*π*=0.0103) and 6 (*π*=0.0093), and the least within lineage 2 (*π*=0.0039) and 3 (*π*=0.0040) strains (Supplementary Table 2). Although spoligotypes tended to cluster within specific clades, there was some evidence of homoplasy, particularly in lineage 4. These anomalies arise from convergent evolution of CRISPR-based spoligotyping polymorphisms^16^. All previously reported lineage-specific RDs^4^ were detected, and their distribution was consistent with clades in the SNP-based phylogeny. There was no evidence of homoplasy events using large sequence polymorphisms, further demonstrating their robustness as phylogenetic markers.

As expected, isolates from lineage 1 harboured the RD239 deletion and had EAI-like spoligotypes. Two natural sublineages designated 1.1 and 1.2 contained distinctive spoligotype compositions (Supplementary Data 1). The Beijing-specific RD105 deletion was restricted to lineage 2, whilst others (RD207, RD181, RD150 and RD142) were observed downstream from the common ancestor (Fig. 1), defining sublineages within lineage 2.2. Isolates belonging to lineage 3 harboured the CAS-specific RD750 deletion, and included sublineages for CAS1-Delhi (33.9%), CAS1-Kili (53.4%), CAS (6.3%) and CAS2 (5.3%) spoligotypes. All non-CAS1-Delhi samples were grouped into the same clade (sublineage 3.1), which was subdivided into two clades harbouring CAS1-Kili and CAS/CAS2 samples, respectively. The Euro-American lineage has been the most poorly characterized historically^21^, and as expected using spoligotypes there was evidence of non-homogeneous sublineages (in particular T, H and LAM families), potentially due to homoplasy events^21^. The phylogeny revealed 36 distinctive clades for lineage 4. All 10 Euro-American lineage RDs were consistently located within one of these clades, further subdivision was achieved and clades with unreported RD were identified (for example, sublineages 4.2, 4.4, 4.7 and 4.9; Fig. 1). Representatives of Haarlem (sublineage 4.1.2.1), Cameroon (4.6.2), LAM (4.3), S-type (4.4.1.1), TUR (4.2.2.1), Uganda (4.6.1), Ural (4.2.1) and X-type (4.1.1) strains were all identified (Supplementary Data 1). Consistent with previous phylogenetic studies, strains belonging to *M. africanum* were split into West-African lineages 1 and 2, where the latter is phylogenetically closer to the *M. bovis* lineage. Members of the recently described phylogenetic lineage 7 were located as expected at an intermediate location between the ancient and modern lineages (Fig. 1).

### Identification of strain-specific SNPs and minimal SNP set

From the 91,648 SNPs, 6,915 lineage and sublineage informative markers were identified (list available at http://pathogenseq.lshtm.ac.uk/tbmolecularbarcode). The distribution of functional categories of genes containing the 91,648 and 6,915 SNP sets did not differ (Supplementary Fig. 2a). Using the informative SNPs (*n*=6,915), there was some evidence of difference in the distribution of functional categories between lineages, namely a greater proportion of lipid metabolism non-synonymous polymorphism in lineage 2 (Supplementary Fig. 2b). Only 88 SNPs were found in drug resistance candidate regions (two promoters, 21 genes) (Supplementary Table 3). Twenty-two non-synonymous SNPs were found in 16 *M. tuberculosis* antigenic genes with known epitopes (Supplementary Table 4).

We focussed on 413 (6%) robust SNPs in essential genes with mutations that lead to synonymous amino acid changes, therefore less likely to be under selective pressure (Supplementary Fig. 2a). Redundancy of markers was observed for most of the clades, and we randomly selected one representative per group, leading to a minimum set of 62 SNPs for MTBC classification (Supplementary Table 5). Reconstruction of a phylogenetic tree using the 62 SNPs for all 1,601 samples resulted in a tree with the same number of delineated clades (Supplementary Fig. 3).

To validate the selection, the 62 SNP classification system was applied to 27 complete reference genomes representing all MTBC lineages and *M. bovis*, when it was found to predict 100% their reported strain types (Supplementary Table 6). Furthermore, we used our scheme to classify 850 samples from Samara, Russia, not included in the 1,601 samples, and found the same reported lineage proportions^22^. More importantly, unclassified samples from lineage 4 in this study could be assigned to Euro-American sublineages by our barcode. A few probable cases of mixed infections were also identified, all combinations of common circulating strain types in that population (Supplementary Table 7).

We investigated lineage-informative SNP sets previously proposed, denoted here as Filliol45 (45 SNPs^21^), Comas93 (93 SNPs^16^) and Homolka71 (71 SNPs^17^). The proportion of these SNPs found among our phylogenetic informative sets differed (Filliol45 29%; Comas93 76%; Homolka71 49%) and some of them were non-segregating across the 1,601 samples (Filliol45 17.8%, Comas93 4.3%; Homolka71 39.0%). Comas93 and Homolka71 sets unambiguously separated six of the seven main MTBC lineages and the resolved sublineages were largely compatible with the ones described in this study (Table 1). Still, not all known RD sublineages^4^, particularly for lineage 4, could be resolved using these classification systems. Phylogenetic trees constructed for the 1,601 samples using these SNP sets (Supplementary Figs 4–6) highlighted the lack of resolution at the sublineage level. The proposed set of 62 SNPs are informative for all seven main MTBC lineages, indicating at least parity in performance with RD typing. Further, the superior number of sublineages classified when compared with other SNP systems demonstrates improved strain-type resolution (Table 1). In recent years, MIRU-VNTR typing (variable number of tandem repeats) has replaced spoligotyping as the genotyping method of choice for public health laboratories and epidemiological studies. An online tool is available to convert MIRU values and assign the strain to an appropriate lineage and spoligotype family^23^. Future work may consider using MIRU data to define sublineages, but the *in silico* determination of the repeats from the short sequencing reads obtained from current high throughput sequencing technology is computationally difficult.

## Discussion

In conclusion, using the whole-genome sequences of over 1,600 MTBC isolates, we characterize a high-resolution map of polymorphisms consisting of more than ninety thousand SNPs. This genomic variation is used to infer phylogenetic relationships both inter- and intra-lineage to an unprecedented level of resolution, and lead to the development of an extendable nomenclature for sublineages. We identify a panel of 62 robust SNP markers (of 413 suitable alternatives) that can be used to construct high resolution and reproducible phylogenies, which can be incorporated in diagnostic assays and assess genotype–phenotype associations. Future work should focus on other types of lineage-specific polymorphisms (for example, insertions, deletions and large structural variants), which are less common than SNPs, but may have major functional consequences. The proposed system has the flexibility to incorporate novel strain types should they be reported. Incorporating anti-tubercular drug-resistance loci will further enhance the usefulness of the barcode as an important tool for tuberculosis control and elimination activities worldwide.

## Methods

### Raw data and sequence analysis

The raw sequence data of 1,804 MTBC isolates available in the public domain were downloaded from the European Nucleotide Archive (ENA; http://www.ebi.ac.uk/ena/). The dataset consists of nine independent whole-genome sequencing studies, available under ENA accessions ERP000192, ERP000276, ERP000520, ERP001731, ERP000111, SRP002589, ERP002611 and ERP000436 (ref. 24), SRA065095 (ref. 25), ERP001885 (ref. 26) and ERP001567 (ref. 5) (Supplementary Table 1). The analysis of the raw sequence data used approaches outlined previously^24^. In brief, all isolate sequence data were mapped to the H37Rv reference genome (Genbank accession number: NC_000962.3) using *BWA*^27^. *SAMtools/BCFtools*^28^ and *GATK*^29^ were employed to call SNPs and mappability values^30^ used to filter out non-unique SNP sites as described in ref. 24 resulting in 91,648 SNP sites. Isolates having less than 15% SNP missing calls were retained (*n*=1,601).

### Phylogenetic analysis and *in silico* genotyping

All samples were *in silico* spoligotyped from raw sequence files (fastq format) using SpolPred software^31^. Coordinates of RDs were identified from ref. 4. Reads covering RDs (+/− 300 bp) were extracted from alignment files (bam format) and subsequently *de novo* assembled using Velvet^32^ into longer sequences called contigs. These contigs were mapped back to the reference genome. If a mapped contig was split into two parts, with high sequence similarity (>95%) and a gap of length equal to the expected RD length, the presence of the RD deletion was reported^33^.

The best-scoring maximum likelihood phylogenetic tree was computed using RAxML v7.4.2 (ref. 34) based on 91,648 sites spanning the whole genome. Given the considerable size of the dataset (1,601 samples × 91,648 SNP sites), the rapid bootstrapping algorithm (*N*=100, *x*=12,345) combined with maximum likelihood search was chosen to construct the phylogenetic tree. The resulting tree was rooted on *M. canettii* (Genbank accession number: NC_019950.1) and nodes were annotated. Subsequently, the ancestral sequence at all internal nodes was computed using DnaPars from the Phylip package^35^. The main lineage- and sublineage-defining nodes were initially identified from the tree, based on the spoligotype and RDs present in each clade. For example, the lineage 1 clade consisted of samples with EAI spoligotypes and RD239. Bootstrap values were computed to assess the confidence of each clade and ensure that all lineage-defined nodes were highly supported (95–100%). For comparison, *RAxML* trees were constructed for the 1,601 samples from alignments using other proposed lineage-informative SNPs (Filliol45, Comas93 and Homolka71; Supplementary Figs 3–6).

### Identification of clade-specific SNPs and minimal SNP set

For each lineage and sublineage, the dataset was split into two populations: one containing all samples descending the clade-defining node and the other with remaining samples. The *F*_ST_ measure was then calculated for each SNP to identify markers with complete between-population allele differentiation (*F*_ST_ >0.99). Similarly, the ancestral reconstructed sequence for the clade-defining node was compared with its closest ancestral node, and the SNP differences derived. A high-confidence set of clade-specific SNPs was obtained by selecting those at the clade-defining internal node and having *F*_ST_ values of >0.99 in between group comparisons. To ensure that clade-specific SNPs were also suitable markers for their use in strain typing assays, we applied filtering criteria: (1) only synonymous SNPs were retained as they are generally under lower selection pressure; (2) SNPs at non-coding regions were discarded since insertions or deletions (indels) are usually more frequent. The density of small indels and large deletions is five and 17 times smaller, respectively, in coding regions of the genome compared to non-coding^24^; and (3) we used only essential genes^20^. The set of drug resistance polymorphism was compiled from TBDreamDB ( www.tbdreamdb.com) and recent studies^25^. The list of known epitopes in H37Rv was extracted from the Immune Epitope Database ( www.iedb.org). The gene functional categories were extracted from Tuberculist (tuberculist.epfl.ch).

## Author contributions

F.C. and T.G.C. conceived the project. J.A.G.-A., J.R.G., J.P., M.V., I.P. and A.P. contributed to the construction of data. F.C. analysed the data. R.M., N.M. and T.G.C. jointly supervised the research. F.C., R.M. and T.G.C. wrote the paper.

## Additional information

**How to cite this article:** Coll, F. *et al.* A robust SNP barcode for typing *Mycobacterium tuberculosis* complex strains. *Nat. Commun.* 5:4812 doi: 10.1038/ncomms5812 (2014).

## Supplementary Material

Supplementary Figures, Supplementary Tables and Supplementary References Supplementary Figures 1-6, Supplementary Tables 1-7 and Supplementary References

Supplementary Data 1 Mycobacterium tuberculosis complex lineages and sub-lineages

## Figures and Tables

**Table 1:** A comparison of SNP-typing systems.

**SNP set and reference**	**Lineage classified (Number of sublineages classified)**									**No. of RD lineages covered**
	**1**	**2**	**3**	**4**	**5**	**6**	**7**	***M. bovis***	**Total**	
This study62 SNPs	Yes (8*)	Yes (6*)	Yes (5*)	Yes (36*)	Yes (0)	Yes (0)	Yes (0)	Yes (0)	7 (55)	19†
Homolka *et al.*^17^71 SNPs	Yes (1‡)	Yes (0)	Yes (0)	Yes (7§)	Yes (2||)	Yes (0)	No (−)	Yes (0)	6 (10)	NA
Comas *et al.*^16^93 SNPs	Yes (1‡)	Yes (2¶)	Yes (0)	Yes (5#)	Yes (0)	Yes (0)	No (−)	Yes (0)	6 (7)	9**
Filliol *et al.*^21^45 SNPs	No (−)	No (−)	No (−)	No (−)	No (−)	No (−)	No (−)	No (−)	6 (−)	NA

NA, not applicable; RD, regions of difference; SNP, single-nucleotide polymorphism.

^*^See Supplementary Data 1 for a complete description of lineages and sublineages.

^†^RD239, 105, 207, 181, 150, 142, 750, 182, 183, 193, 122, 726, 219, 761, 115, 174, 724, 711, 702; 239.

^‡^Lineage 1.2.1.

^§^Lineages 4.6.2.2, 4.1.2.1, 4.3, 4.4.1.1, 4.2.2.1, 4.2.1 and an ambiguous ‘Ghana’.

^||^West Africanum Ia and Ib.

^¶^Lineages 2.1 and 2.2.

^#^Lineages 4.6.2.2, 4.6.1, 4.1.1, 4.1.2.1 and 4.3.

^**^RD105, 207, 750, 726, 724, 182, 711, 702 and 7.
