# Supplementary material for: A robust SNP barcode for typing Mycobacterium tuberculosis complex strains
Source: Nat Commun. 2014 Sep 1;5:4812. doi: 10.1038/ncomms5812 (PMC4166679; doi:10.1038/ncomms5812)
Supplement: Supplementary Figures, Supplementary Tables and Supplementary References — Supplementary Figures 1-6, Supplementary Tables 1-7 and Supplementary References [file ncomms5812-s1.pdf]

Supplementary Figure 1

(A) Summary of functional categories for 3 sets of SNPs: all (n=91648, left), lineage-specific (n=6915, middle) and filtered/diagnostic lineage-specific (n=413, right) (B) Lineage-specific SNPs distribution for each of the main six MTBC lineages across gene functional categories (1-7).

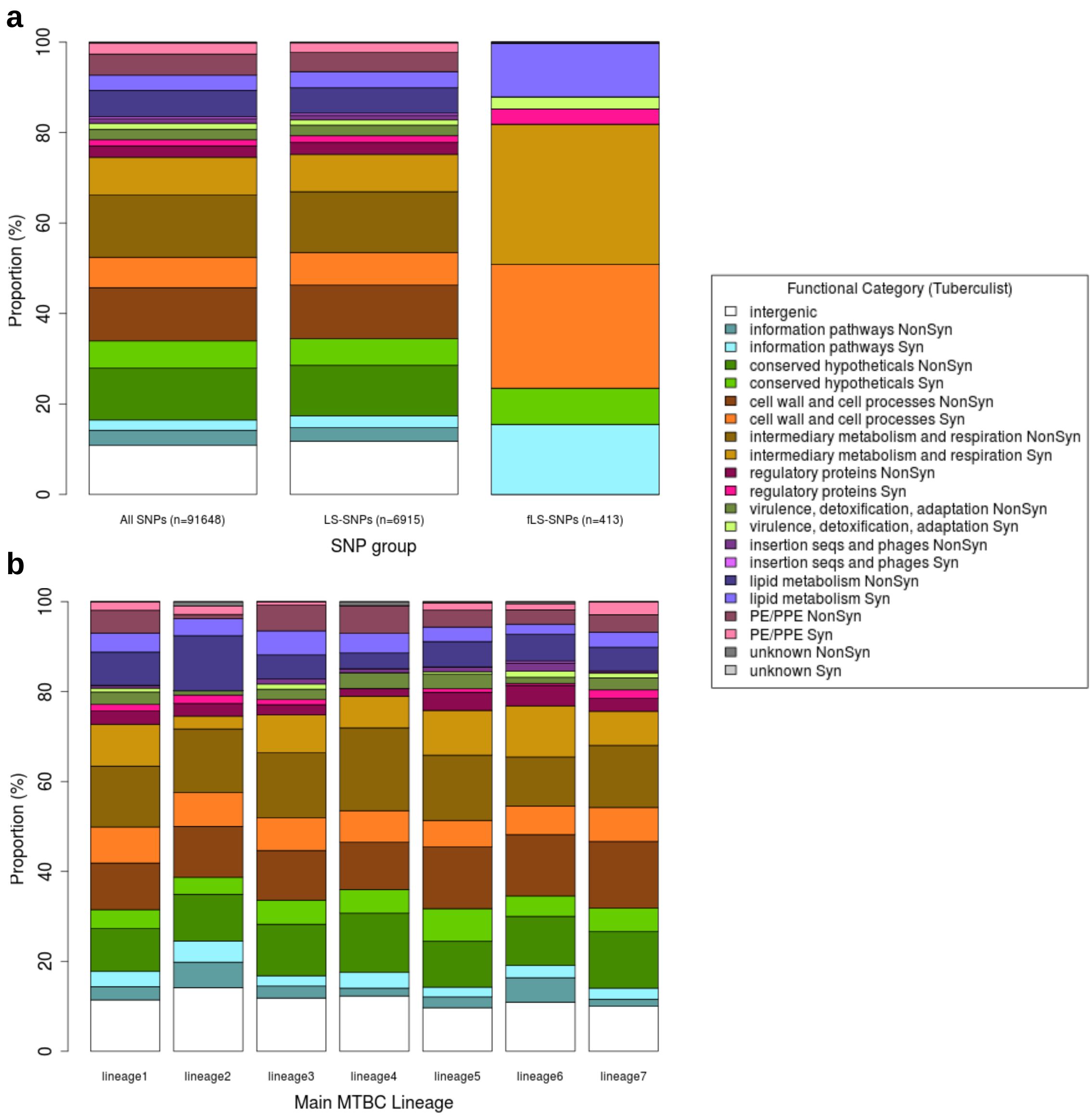

Supplementary Figure 2

SNP Allele Frequency Spectrum

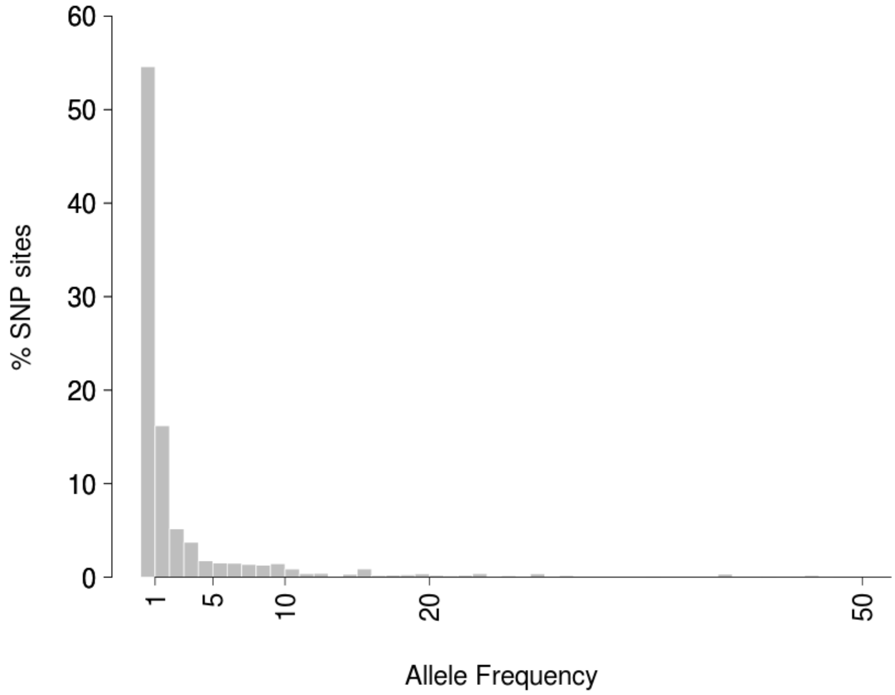

Supplementary Figure 3

Global phylogeny constructed using the proposed minimum set of 62 SNPs separates all 1601 samples into their corresponding lineage and sub-lineage.

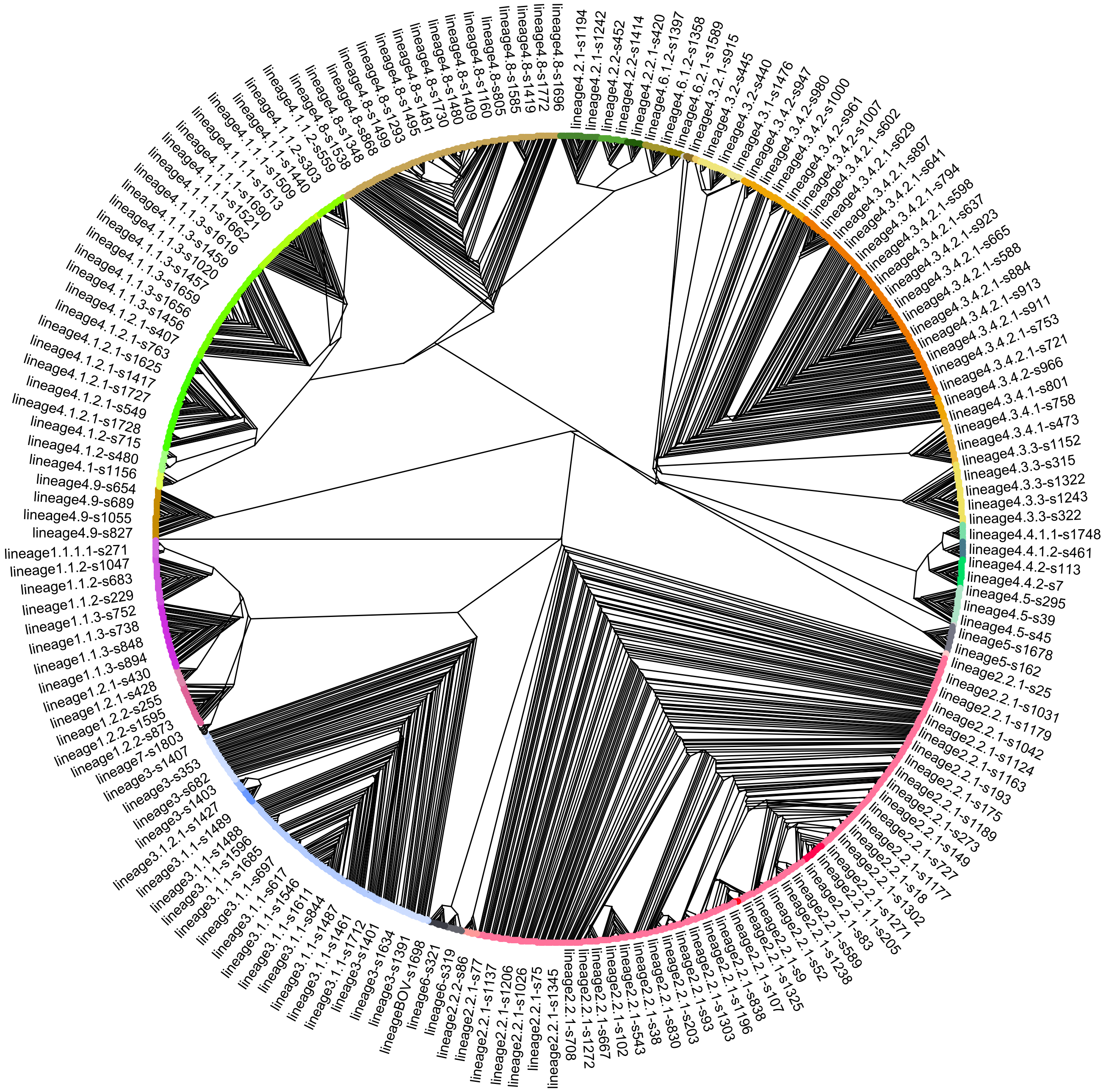

Supplementary Figure 4

Global phylogeny constructed using the set of 45 SNPs proposed by [24] yielded an incompatible classification compared to that obtained by Comas93 (Supplementary Figure 5) and Homolka71 (Supplementary Figure 6). The MTBC lineages could not be unambiguously separated (lineages 1, 5, 6 and *M. bovis*) or were spread across multiple clades (lineages 2, 3 and 4).

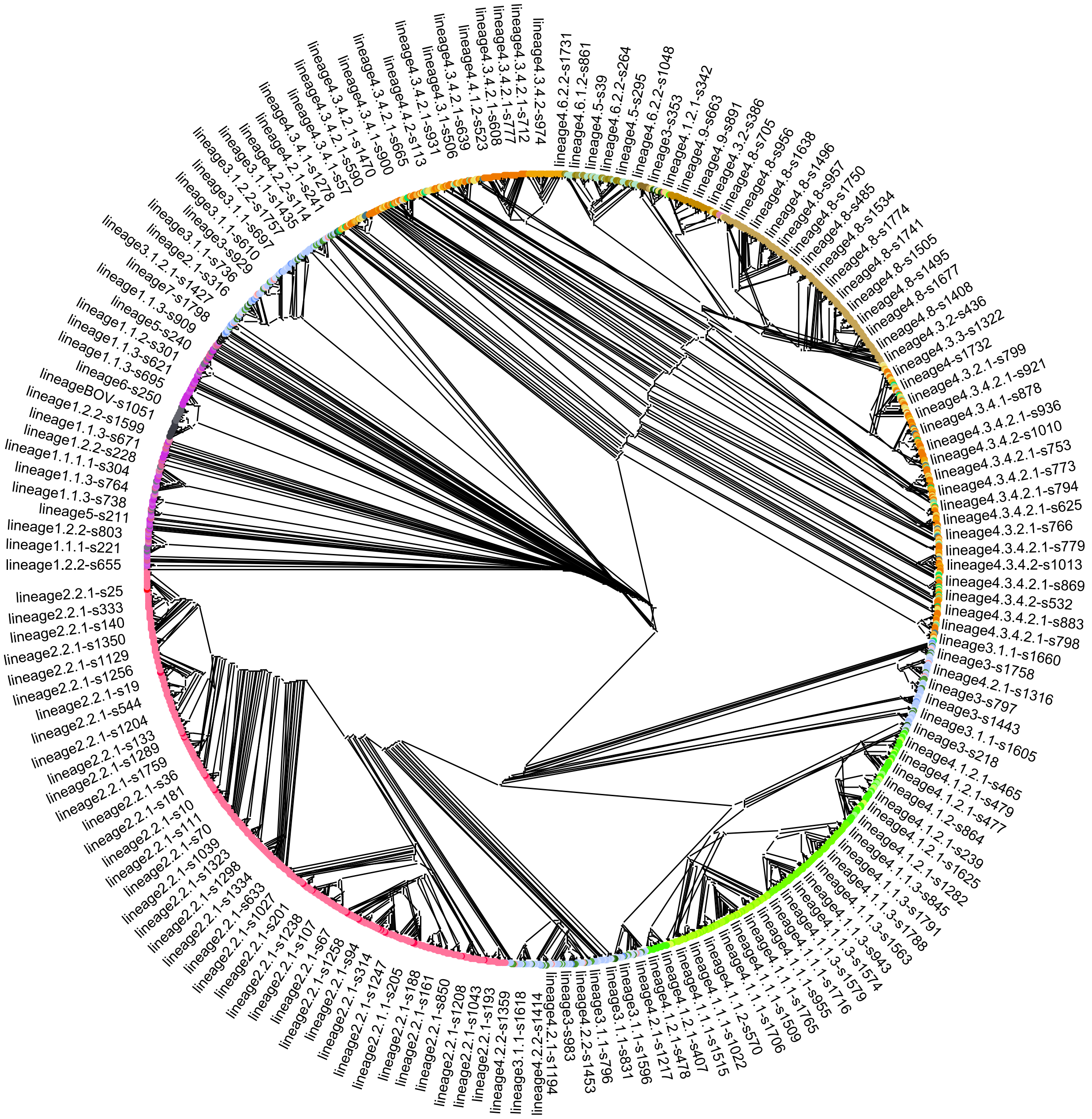

Supplementary Figure 5

A global phylogeny constructed using the 93 lineage-specific SNPs proposed by [25] shows all 6 MTBC main lineages and *M. bovis* unambiguously separated in different clades. Samples from sub-lineage 2.1 (non-Beijing), 2.2 (Beijing), 4.3 (LAM), 4.1.1 (X-family), 4.1.2.1 (Haarlem), 4.6.2.2 (Cameroon) and 4.6.1 (Uganda) were all constrained to specific clades. However, the markers lacked resolution at a sub-lineage level, with the majority being unresolvable.

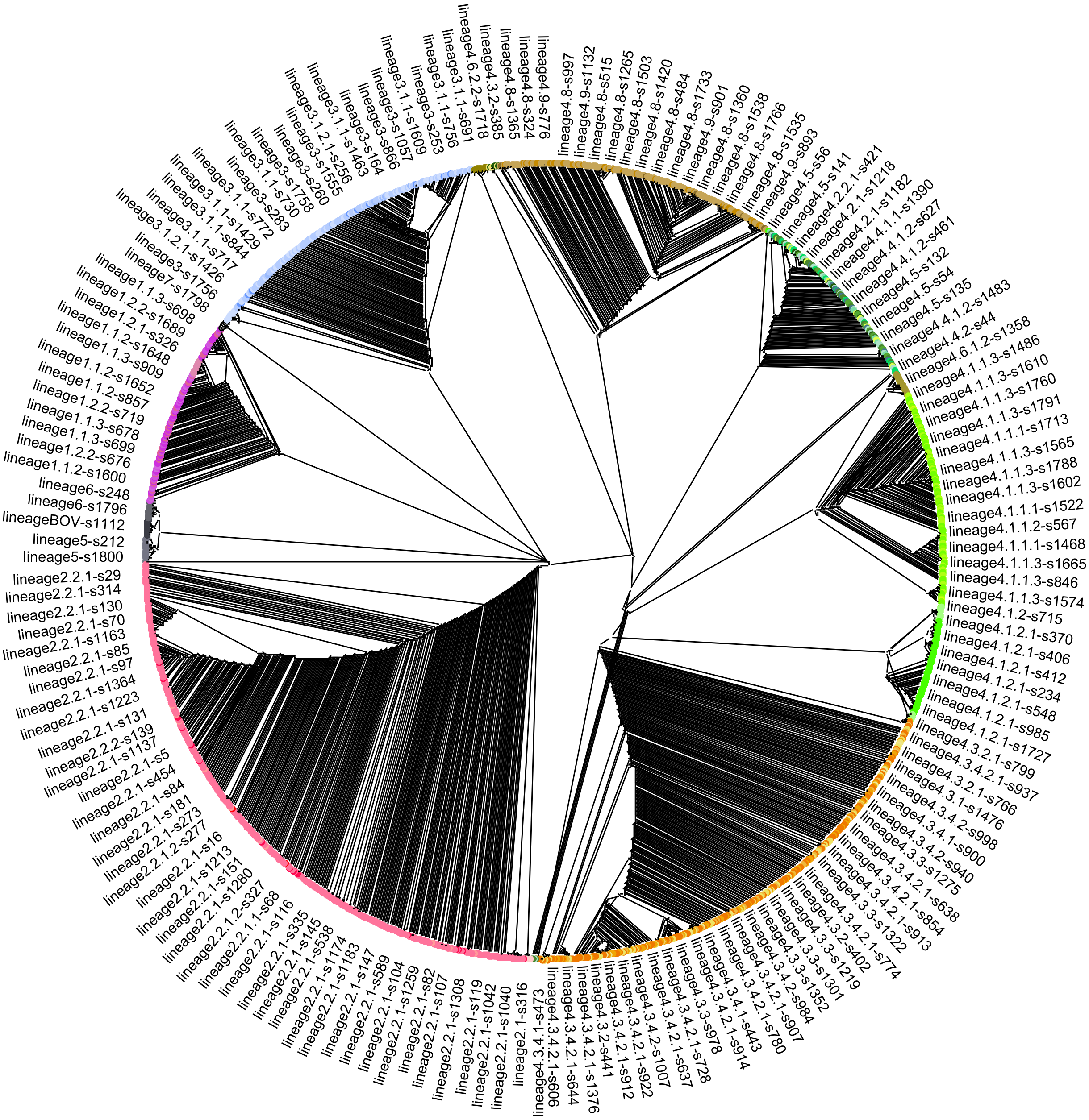

Supplementary Figure 6

Global phylogeny constructed using the 71 phylogenetically informative SNPs proposed by [26]. The phylogeny is largely congruent with that built using Comas93 SNP set; all main seven MTBC lineages are clearly separated. Samples belonging to sub-lineages 4.2.1 (Ural), 4.2.2.1 (TUR), 4.3 (LAM), 4.4.1.1 (S-type), 4.1.2.1 (Haarlem) and 4.6.2.2 (Cameroon) are all restricted to specific clades. However other sub-lineages (particularly from lineage 4) were not congruent with the RD system with some samples with the same RD (e.g. RD115 for 4.3.3 sub-lineage) spread across different clades.

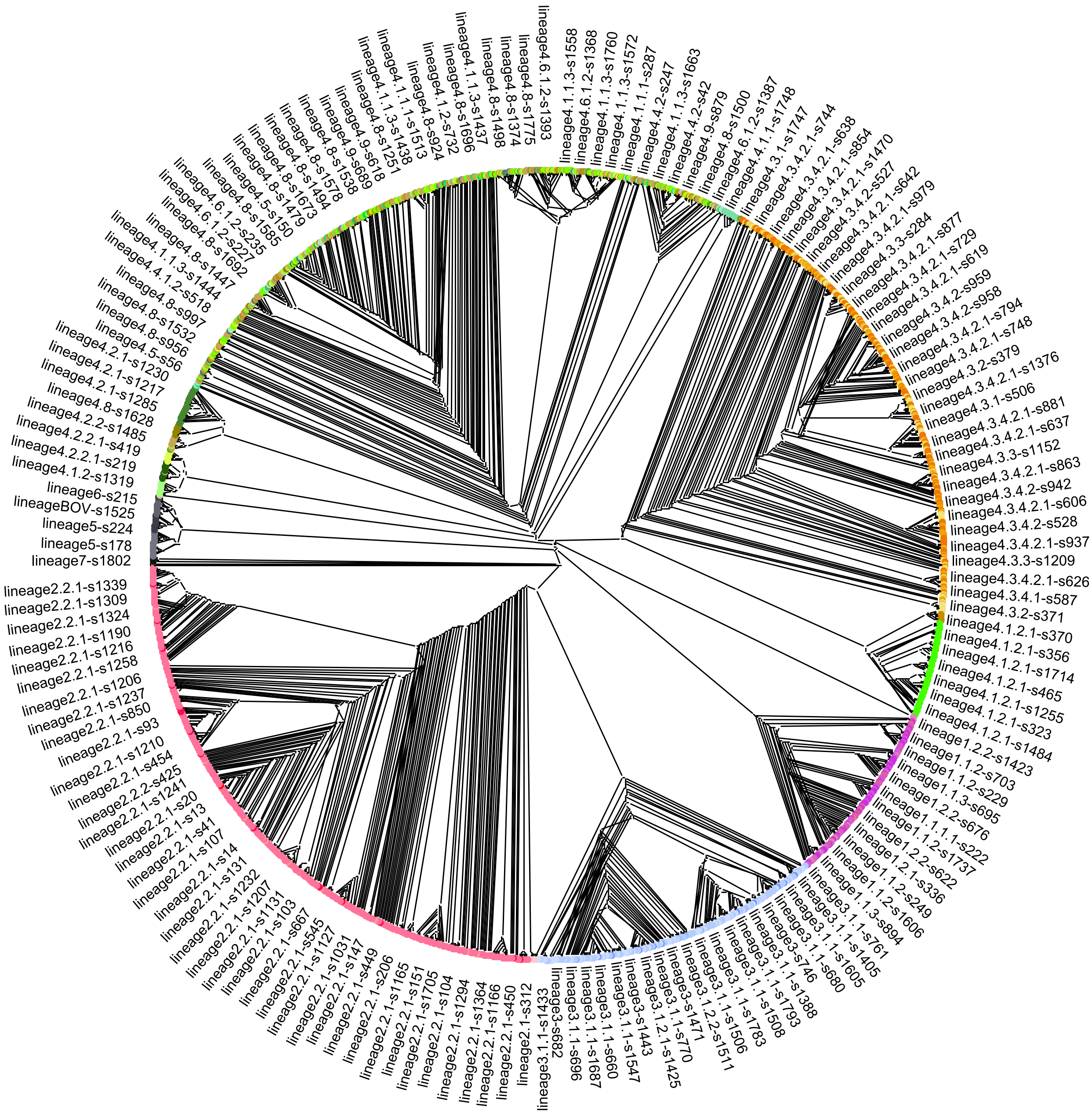

Supplementary Table 1

The 1601 MTBC isolates in this study and their lineages

**Study***	***n***	**# SNPs**	**Lin. 1 %**	**Lin. 2 %**	**Lin. 3 %**	**Lin. 4 %**	**Lin. 5 %**	**Lin. 6 %**	**Lin. 7 %**	***M. bovis %***
Canada	19	1,021	0	0	0	100	0	0	0	0
China[1]	161	19,314	0	75.8	1.2	23.0	0	0	0	0
Global	166	30,770	14.5	34.9	12.7	24.1	7.8	5.4	0	0.6
Malawi	338	19,240	18.9	5.6	14.8	60.7	0	0	0	0
Netherlands	125	8,635	4.0	16.8	0.8	78.4	0	0	0	0
Portugal	81	7,163	0	6.2	1.2	92.6	0	0	0	0
Russia	259	18,699	1.5	58.7	1.9	35.9	0.4	0	0	1.5
Uganda	51	8,019	2.0	2.0	27.5	68.6	0	0	0	0
UK	390	19,408	5.9	2.6	24.4	65.1	0.5	0	0	1.5
Ethiopia[2]	4	2345	0	0	0	0	0	0	100	0
Djibouti[3]	7	5445	0	28.6	0	0	14.3	28.6	28.6	0
Overall	**1,601**	**91648**	***7.5***	***24.4***	***11.8***	***53.4***	***1.1***	***0.7***	***0.4***	***0.7***

* See [4]

Supplementary Table 2

Lineage characteristics

**Characteristics**	**1**	**2**	**3**	**4**	**5**	**6**	**7**
n	121	390	189	856	17	11	6
Description	East-African-Indian	East-Asian	Indo-Oceanic	Euro-American	West Africa I<br>*M. africanum I*	West Africa 2<br>*M. africanum* 2	Lineage 7
Spoligotypes	EAI	Beijing	CAS	T, H, LAM, X, S	AFRI_2, AFRI_3	AFRI_1	Unknown
Total SNPS	14,661	22,490	9864	38,033	4761	4560	2458
Avg. SNPs/ genome	1970	1322	1314	746	1955	2064	1962
(range)	(1573 - 2094)	(1041 - 1403)	(1046 - 1389)	(15-962)	(1831-2030)	(1932-2157)	(1921-1997)
Mean SNPs to MRCA	93	69	53	43	185	207	90
Diversity π	0.0102	0.0039	0.0040	0.0077	0.0058	0.0093	0.0035
RDs present	239	105, 207, 181, 150, 142	750	182, 183, 193, 122, 726, 219, 761, 115, 174, 724	711	702	None
No. Sub-lineages	8	6	5	36	0	0	0
No. Informative SNPs	473	106	262	114	372	220	898
Coding SNPs (% NS)	419 (62.3)	91 (69.2)	231 (64.1)	100 (69)	336 (65.2)	196 (58.2)	808 (64.1)

Abbreviations: MRCA: most recent common ancestor; RD: region of difference; NS: non-synonymous

Supplementary Table 3

The set of 62 phylogenetically informative SNPs for MTBC typing

lineage	Position*	Gene coord.	Allele change	Codon number	Codon change	Amino acid change	Locus Id	Gene name
lineage1	615938	1104	G/A	368	GAG/GAA	E/E	Rv0524	*hemL*
lineage1.1	4404247	1056	G/A	352	CTG/CTA	L/L	Rv3915	-
lineage1.1.1	3021283	711	G/A	237	CGG/CGA	R/R	Rv2707	-
lineage1.1.1.1	3216553	339	G/A	113	GTC/GTT	V/V	Rv2907c	*rimM*
lineage1.1.2	2622402	51	G/A	17	GCC/GCT	A/A	Rv2343c	*dnaG*
lineage1.1.3	1491275	1038	G/A	346	CAC/CAT	H/H	Rv1326c	*glgB*
lineage1.2.1	3479545	375	C/A	125	GCC/GCA	A/A	Rv3111	*moaC1*
lineage1.2.2	3470377	303	C/T	101	CAG/CAA	Q/Q	Rv3101c	*ftsX*
lineage2	497491	810	G/A	270	GAC/GAT	D/D	Rv0411c	*glnH*
lineage2.1	1881090	5787	C/T	1929	GGC/GGT	G/G	Rv1661	*pks7*
lineage2.2	2505085	615	G/A	205	GCC/GCT	A/A	Rv2231c	*cobC*
lineage2.2.1	797736	804	C/T	268	CTC/CTT	L/L	Rv0697	-
lineage2.2.1.1	4248115	1602	C/T	534	GAC/GAT	D/D	Rv3795	*embB*
lineage2.2.1.2	3836274	618	G/A	206	TTC/TTT	F/F	Rv3417c	*groEL1*
lineage2.2.2	346693	1059	G/T	353	TCG/TCT	S/S	Rv0284	*eccC3*
lineage3	3273107	894	C/A	298	GCC/GCA	A/A	Rv2936	*drrA*
lineage3.1.1	1084911	840	G/A	280	TAC/TAT	Y/Y	Rv0973c	*accA2*
lineage3.1.2	3722702	930	G/C	310	CTC/CTG	L/L	Rv3336c	*trpS*
lineage3.1.2.1	1237818	375	C/G	125	CTG/CTC	L/L	Rv1111c	-
lineage3.1.2.2	2874344	2142	G/A	714	CGC/CGT	R/R	Rv2555c	*alaS*
lineage4**	931123	171	T/C	57	TAT/TAC	Y/Y	Rv0835	*lpqQ*
lineage4.1	62657	2262	G/A	754	CCG/CCA	P/P	Rv0058	*dnaB*
lineage4.1.1	514245	1077	C/T	359	GTG/GTA	V/V	Rv0425c	*ctpH*
lineage4.1.1.1	1850119	1917	C/T	639	ACG/ACA	T/T	Rv1640c	*lysX*
lineage4.1.1.2	541048	444	T/G	148	TCA/TCC	S/S	Rv0450c	*mmpL4*
lineage4.1.1.3	4229087	741	C/T	247	AAC/AAT	N/N	Rv3782	*glfT1*
lineage4.1.2	891756	514	A/G	172	TTG/CTG	L/L	Rv0798c	*cfp29*
lineage4.1.2.1	107794	195	C/T	65	GCC/GCT	A/A	Rv0098	*fcoT*
lineage4.2	2411730	393	G/C	131	TCC/TCG	S/S	Rv2152c	*murC*
lineage4.2.1	783601	1117	A/C	373	AGG/CGG	R/R	Rv0684	*fusA1*
lineage4.2.2	1487796	636	C/A	212	ATC/ATA	I/I	Rv1324	-
lineage4.2.2.1	1455780	286	T/C	96	TTG/CTG	L/L	Rv1299	*prfA*
lineage4.3	764995	1626	C/G	542	GCC/GCG	A/A	Rv0668	*rpoC*
lineage4.3.1	615614	780	C/A	260	GCC/GCA	A/A	Rv0524	*hemL*
lineage4.3.2	4316114	483	G/A	161	GCC/GCT	A/A	Rv3843c	-
lineage4.3.2.1	3388166	705	C/G	235	ACG/ACC	T/T	Rv3029c	*fixA*
lineage4.3.3	403364	2478	G/A	826	CCC/CCT	P/P	Rv0338c	-
lineage4.3.4	3977226	165	G/A	55	TTG/TTA	L/L	Rv3538	-
lineage4.3.4.1	4398141	1545	G/A	515	TCG/TCA	S/S	Rv3910	-

lineage4.3.4.2	1132368	744	C/T	248	ACC/ACT	T/T	Rv1013	*pks16*
lineage4.3.4.2.1	1502120	522	C/A	174	ACC/ACA	T/T	Rv1333	-
lineage4.4	4307886	1029	G/A	343	CGC/CGT	R/R	Rv3834c	*serS*
lineage4.4.1	4151558	660	G/A	220	GGC/GGT	G/G	Rv3708c	*asd*
lineage4.4.1.1	355181	684	G/A	228	AAG/AAA	K/K	Rv0291	*mycP3*
lineage4.4.1.2	2694560	405	G/C	135	CTC/CTG	L/L	Rv2397c	*cysA1*
lineage4.4.2	4246508	3276	G/A	1092	GCG/GCA	A/A	Rv3794	*embA*
lineage4.5	1719757	1032	G/T	344	CCG/CCT	P/P	Rv1524	-
lineage4.6	3466426	666	G/A	222	GTC/GTT	V/V	Rv3097c	*lipY*
lineage4.6.1	4260268	879	G/C	293	GCC/GCG	A/A	Rv3800c	*pks13*
lineage4.6.1.1	874787	555	G/A	185	CCG/CCA	P/P	Rv0781	*ptrBa*
lineage4.6.1.2	1501468	543	G/C	181	CCG/CCC	P/P	Rv1332	-
lineage4.6.2	4125058	642	G/C	214	CGG/CGC	R/R	Rv3683	-
lineage4.6.2.1	3570528	684	C/G	228	CGG/CGC	R/R	Rv3198c	*uvrD2*
lineage4.6.2.2	2875883	603	C/T	201	CTG/CTA	L/L	Rv2555c	*alaS*
lineage4.7	4249732	3219	C/G	1073	GCC/GCG	A/A	Rv3795	*embB*
lineage4.8	3836739	153	G/A	51	GAC/GAT	D/D	Rv3417c	*groEL1*
lineage4.9**	1759252	1572	G/T	524	TCG/TCT	S/S	Rv1552	*frdA*
lineage5	1799921	339	C/A	113	GGC/GGA	G/G	Rv1599	*hisD*
lineage6	1816587	399	C/G	133	GTC/GTG	V/V	Rv1617	*pykA*
lineage7	1137518	543	G/A	181	AAC/AAT	N/N	Rv1018c	*glmU*
lineageBOV	2831482	1110	A/G	370	GGT/GGC	G/G	Rv2515c	-
lineageBOV_AFRI	1882180	477	C/T	159	GCC/GCT	A/A	Rv1662	*pks8*

* based on reference NC_000962.3

** In these two cases the reference allele is the one specific to the lineage

Supplementary Table 4 Lineage predictions for a set of reference genomes

Species	Strain	Accession	Predicted lineage; sub-lineage	Known lineage; sub-lineage (reference)
M. africanum	GM041182	NC_015758.1	6; BOV_AFRI	Mycobacterium africanum West African 2 [5]
M. bovis	BCG strain Korea 1168P	CP003900.2	BOV; BOV_AFRI	Mycobacterium bovis BCG [6]
M. bovis	BCG strain Mexico	NC_016804.1	BOV; BOV_AFRI	Mycobacterium bovis BCG [7]
M. bovis	BCG strain Moreau RDJ	AM412059.2	BOV; BOV_AFRI	Mycobacterium bovis BCG [8]
M. bovis	BCG strain Pasteur 1173P2	NC_008769.1	BOV; BOV_AFRI	Mycobacterium bovis BCG [9]
M. bovis	BCG strain Tokyo 172	NC_012207.1	BOV; BOV_AFRI	Mycobacterium bovis BCG [10]
M. tuberculosis	7199-99	NC_020089.1	4;4.1; 4.1.2; 4.1.2.1	Hamburg clone, Haarlem lineage [11]
M. tuberculosis	BT1	CP002883.1	2; 2.2; 2.2.1	Beijing/W
M. tuberculosis	BT2	CP002882.1	2; 2.2; 2.2.1	Beijing/W
M. tuberculosis	CAS NITR204	NC_021193.1	3	CAS strain [12]
M. tuberculosis	CCDC5079	NC_017523.1	2; 2.2; 2.2.1	Beijing family [13]
M. tuberculosis	CCDC5180	NC_017522.1	2; 2.2; 2.2.1	Beijing family [13]
M. tuberculosis	CDC1551	NC_002755.2	4;4.1; 4.1.1; 4.1.1.3	Euro-American lineage strain, X family [14]
M. tuberculosis	CTRI-2	CP002992.1	4;4.3; 4.3.3	Euro-American lineage 4 strain, LAM family, LAM9 [15]
M. tuberculosis	EAI5	CP006578.1	1; 1.1	East African Indian lineage 1, EAI5 [16]
M. tuberculosis	EAI5 NITR206	NC_021194.1	1; 1.1	East African Indian lineage 1, EAI5 [12]
M. tuberculosis	F11	NC_009565.1	4;4.3; 4.3.2; 4.3.2.1	Euro-American lineage 4 strain, LAM family, LAM3 [17]
M. tuberculosis	HKBS1	CP002871.1	2; 2.2; 2.2.1	Beijing/W Lineage
M. tuberculosis	KZN1435	NC_012943.1	4;4.3; 4.3.3	Euro-American lineage 4 strain, LAM family [18]
M. tuberculosis	KZN4207	NC_016768.1	4;4.3; 4.3.3	Euro-American lineage 4 strain, LAM family [18]
M. tuberculosis	KZN605	NC_018078.1	4;4.3; 4.3.3	Euro-American lineage 4 strain, LAM family [18]
M. tuberculosis	RGTB327	CP003233.1	4;4.3; 4.3.4	Not reported [19]
M. tuberculosis	RGTB423	NC_017528.1	1; 1.2.2	Not reported [19]

M. tuberculosis	Strain Beijing NITR203	NC_021054.1	2; 2.2; 2.2.1; 2.2.1.1	Beijing strain [12]
M. tuberculosis	Strain Erdman ATCC35801	AP012340.1	4;4.1; 4.1.2; 4.1.2.1	Euro-American lineage 4 strain, Haarlem strain [20]
M. tuberculosis	Strain Haarlem	CP001664.1	4;4.1; 4.1.2; 4.1.2.1	Euro-American lineage 4 strain, Haarlem strain
M. tuberculosis	UT205	NC016934.1	4;4.3; 4.3.4; 4.3.4.2	Euro-American lineage 4 strain, LAM family [21]

Supplementary Table 5

Lineage-specific SNPs at drug resistance genes

Drug	*Gene name*	Lineage	Position**	Gene Coord.	Allele Change	Codon Number	Codon Change	Amino Acid Change	Locus Tag
INH	*katG*	4	2154724*	1388	C/A	463	CGG/CTG	R/L	Rv1908c
INH	*katG*	BOV_AFRI	2155503	609	G/A	203	ACC/ACT	T/T	Rv1908c
INH	*inhA*	6	1674434	233	T/C	78	GTG/GCG	V/A	Rv1484
INH	*ahpC promoter*	3	2726105*	-	G/A	-	-	-	-
INH	*kasA*	4.3.3	2518919	805	G/A	269	GGT/AGT	G/S	Rv2245
INH	*ndh*	4.4.1	2102990	53	A/G	18	GTG/GCG	V/A	Rv1854c
INH	*ndh*	5	2101921	1122	C/T	374	TCG/TCA	S/S	Rv1854c
INH	*ndh*	7	2102218	825	G/A	275	GTC/GTT	V/V	Rv1854c
EMB	*embA*	1	4245969	2737	C/T	913	CCG/TCG	P/S	Rv3794
EMB	*embA*	1.1	4243848	616	G/A	206	GTG/ATG	V/M	Rv3794
EMB	*embA*	1.2.1	4244420	1188	G/C	396	GTG/GTC	V/V	Rv3794
EMB	*embA*	2.1	4246088	2856	A/G	952	CAA/CAG	Q/Q	Rv3794
EMB	*embA*	2.2	4243460*	228	C/T	76	TGC/TGT	C/C	Rv3794
EMB	*embA*	4.4.2	4246508	3276	G/A	1092	GCG/GCA	A/A	Rv3794
EMB	*embA*	4.6.1.2	4245055	1823	C/A	608	ACC/AAC	T/N	Rv3794
EMB	*embB*	2.2.1.1	4248115	1602	C/T	534	GAC/GAT	D/D	Rv3795
EMB	*embB*	4.1.1.2	4246930	417	G/C	139	CAG/CAC	Q/H	Rv3795
EMB	*embB*	4.4.1.2	4249012	2499	G/A	833	CTG/CTA	L/L	Rv3795
EMB	*embB*	4.7	4249732	3219	C/G	1073	GCC/GCG	A/A	Rv3795
EMB	*embB*	7	4248073	1560	C/T	520	ACC/ACT	T/T	Rv3795
EMB	*embB*	BOV_AFRI	4246864	351	C/T	117	GTC/GTT	V/V	Rv3795
EMB	*embC*	1	4241042	1180	A/G	394	AAC/GAC	N/D	Rv3793
EMB	*embC*	3	4242075*	2213	G/A	738	CGG/CAG	R/Q	Rv3793
EMB	*embC*	3.1.1	4241562	1700	G/A	567	CGC/CAC	R/H	Rv3793
EMB	*embC*	4.1	4242803*	2941	G/C	981	GTG/CTG	V/L	Rv3793
EMB	*embC*	4.1.1.1	4240897	1035	C/G	345	CGC/CGG	R/R	Rv3793
EMB	*embC*	4.6.2.1	4242883	3021	C/T	1007	CCC/CCT	P/P	Rv3793
EMB	*embC*	4.9	4242643	2781	C/T	927	CGC/CGT	R/R	Rv3793

EMB	*embC*	7	4240153	291	G/A	97	TCG/TCA	S/S	Rv3793
EMB	*embR*	1	1417019	329	C/T	110	TGC/TAC	C/Y	Rv1267c
EMB	*embR*	4.6.1.2	1416410	938	A/C	313	CTG/CGG	L/R	Rv1267c
EMB	*embR*	4.6.2.1	1416702	646	A/G	216	TAC/CAC	Y/H	Rv1267c
EMB	*embR*	7	1416977	371	T/C	124	CAC/CGC	H/R	Rv1267c
EMB	*ubiA*	4.4.2	4268928	906	G/A	302	GGC/GGT	G/G	Rv3806c
EMB	*ubiA*	4.4.2	4269375	459	C/T	153	GTG/GTA	V/V	Rv3806c
EMB	*ubiA*	BOV_AFRI	4269351	483	G/A	161	GCC/GCT	A/A	Rv3806c
EMB	*aftA*	1.2.2	4238120	189	G/A	63	CAG/CAA	Q/Q	Rv3792
EMB	*aftA*	4.1.2.1	4239298	1367	C/T	456	GCC/GTC	A/V	Rv3792
EMB	*aftA*	4.4	4238963	1032	C/T	344	CAC/CAT	H/H	Rv3792
EMB	*aftA*	5	4239843	1912	A/C	638	AAG/CAG	K/Q	Rv3792
EMB	*aftA*	7	4238778	847	G/A	283	GTG/ATG	V/M	Rv3792
EMB	*nuoD*	2.1	3513538	201	A/T	67	GAA/GAT	E/D	Rv3148
EMB	*nuoD*	4.3.4.2	3514512	1175	G/C	392	GGT/GCT	G/A	Rv3148
RMP	*rpoB*	3	762434	2628	T/G	876	GGT/GGG	G/G	Rv0667
RMP	*rpoB*	4	763031	3225	T/C	1075	GCT/GCC	A/A	Rv0667
RMP	*rpoC*	1	763884	515	C/T	172	GCC/GTC	A/V	Rv0668
RMP	*rpoC*	1	763886	517	C/A	173	CGG/AGG	R/R	Rv0668
RMP	*rpoC*	1.1	765171	1802	C/T	601	CCG/CTG	P/L	Rv0668
RMP	*rpoC*	1.1.3	765230	1861	G/A	621	GCG/ACG	A/T	Rv0668
RMP	*rpoC*	4.1	765150	1781	G/A	594	GGG/GAG	G/E	Rv0668
RMP	*rpoC*	4.3	764995	1626	C/G	542	GCC/GCG	A/A	Rv0668
RMP	*rpoC*	7	764013	644	A/C	215	GAG/GCG	E/A	Rv0668
RMP	*rpoC*	7	766955	3586	G/A	1196	GAG/AAG	E/K	Rv0668
STR	*rrs*	4.3.2	1472337	-	C/T	-	-	-	-
STR	*gid*	1	4407873	330	C/A	110	GTG/GTT	V/V	Rv3919c
STR	*gid*	1.1.3	4407780	423	C/T	141	GCG/GCA	A/A	Rv3919c
STR	*gid*	2.2	4407927	276	T/G	92	GAA/GAC	E/D	Rv3919c
STR	*gid*	4	4407588	615	T/C	205	GCA/GCG	A/A	Rv3919c
STR	*gid*	4.3	4408156	47	A/C	16	CTT/CGT	L/R	Rv3919c
FLQ	*gyrA*	1	8452	1151	C/T	384	GCA/GTA	A/V	Rv0006
FLQ	*gyrA*	1.2.1	9260	1959	G/C	653	CTG/CTC	L/L	Rv0006
FLQ	*gyrA*	3.1.2.2	9611	2310	C/T	770	GAC/GAT	D/D	Rv0006
FLQ	*gyrA*	4.3.3	8040	739	G/A	247	GGC/AGC	G/S	Rv0006
FLQ	*gyrA*	4.5	7892	591	G/A	197	CTG/CTA	L/L	Rv0006
FLQ	*gyrA*	4.6.1	7539*	238	A/G	80	ACC/GCC	T/A	Rv0006

FLQ	*gyrA*	5	9566	2265	C/T	755	TAC/TAT	Y/Y	Rv0006
FLQ	*gyrA*	7	8876	1575	C/T	525	TAC/TAT	Y/Y	Rv0006
FLQ	*gyrB*	1	6112	873	G/C	291	ATG/ATC	M/I	Rv0005
FLQ	*gyrB*	1.1.2	6124	885	C/T	295	GCC/GCT	A/A	Rv0005
FLQ	*gyrB*	4.3.2.1	5520	281	C/T	94	CCG/CTG	P/L	Rv0005
FLQ	*gyrB*	4.3.2.1	7222	1983	C/T	661	AGC/AGT	S/S	Rv0005
PZA	*rpsA*	2	1834177*	636	A/C	212	CGA/CGC	R/R	Rv1630
PZA	*rpsA*	7	1834916	1375	A/C	459	ACC/CCC	T/P	Rv1630
ETH	*ethA*	1.2.2	4326439	1035	G/T	345	AAC/AAA	N/K	Rv3854c
ETH	*ethA*	3.1.2.2	4326176	1298	T/G	433	GAG/GCG	E/A	Rv3854c
ETH	*ethA*	4.6.2.2	4326739	735	G/C	245	CGC/CGG	R/R	Rv3854c
ETH	*ethR*	4.6.2.2	4328004	456	G/A	152	GTG/GTA	V/V	Rv3855
ETH	*inhA*	6	1674434	233	T/C	78	GTG/GCG	V/A	Rv1484
AMI	*rrs*	4.3.2	1472337	-	C/T	-	-	-	-
AMI	*gid*	1	4407873*	330	C/A	110	GTG/GTT	V/V	Rv3919c
AMI	*gid*	1.1.3	4407780	423	C/T	141	GCG/GCA	A/A	Rv3919c
AMI	*gid*	2.2	4407927*	276	T/G	92	GAA/GAC	E/D	Rv3919c
AMI	*gid*	4	4407588*	615	T/C	205	GCA/GCG	A/A	Rv3919c
AMI	*gid*	4.3	4408156*	47	A/C	16	CTT/CGT	L/R	Rv3919c
CAP	*rrs*	4.3.2	1472337	-	C/T	-	-	-	-
KAN	*rrs*	4.3.2	1472337	-	C/T	-	-	-	-
AMK	*rrs*	4.3.2	1472337	-	C/T	-	-	-	-
CAP	*tlyA*	7	1918281	342	A/C	114	GGA/GGC	G/G	Rv1694

Abbreviations: RMP: rifampicin; INH: isoniazid; STR: streptomycin; ETH: ethionamide; EMB: ethambutol; PZA: pyrazinamide; AMI: Aminoglycosides; AMK: amikacin; CAP: capreomycin; KAN: kanamycin; FLQ: fluoroquinolones; MOX: moxifloxacin; OFX: ofloxacin; PAS: para-aminosalicylic acid; NS: non-synonymous; S: synonymous; I: intergenic; Ref: reference; Alt: alternative; NT: nucleotide. **based on reference NC_00096.3

* Also described in [22]

Supplementary Table 6

Non-synonymous lineage-specific SNPs at known epitopes in H37Rv

Epitope Id	Start	End	Locus Id	Amino acid sequence	Lineage_SNP
7000006195549910	157070	157129	Rv0129c	AAVGLSMSGGSALILAAYYP	3_157129
7000006195549960	2952682	2952741	Rv2626c	DDDRLHGMLTDRDIVIKGLA	7_2952738
7000006195549960	2952712	2952771	Rv2626c	DRDIVIKGLAAGLDPNTATA	7_2952738
7000006195549960	2955088	2955147	Rv2628	IRAVGPYAWAGRCGRIGRWG	7_2955128
7000006195549970	2955358	2955417	Rv2628	DWPAAYAIGEHLSVEIAVAV	7_2955392
7000006195549970	2955058	2955117	None	MSTQRPRHSGIRAVGPYAWA	4.8_2955061
7000006195549970	2955118	2955177	Rv2628	GRCGRIGRWGVHQEAMMNLA	7_2955128
7000006195549980	1403228	1403287	Rv1255c	RRARWVVRMLTSLLMFPGRD	5_1403266
7000006195550000	3079639	3079698	Rv2770c	VANRALLAELTATNILGQNV	1.1.1.1_3079685
7000006195550000	3079669	3079728	Rv2770c	TATNILGQNVSAIAATEARY	1.1.1.1_3079685
7000006195550000	3079819	3079878	Rv2770c	SHITNPAGLAHQAAAVGQAG	4_3079877
7000006195550020	2955058	2955102	None	MSTQRPRHSGIRAVG	4.8_2955061
7000006195550020	2955088	2955132	Rv2628	IRAVGPYAWAGRCGR	7_2955128
7000006195550020	2955103	2955147	Rv2628	PYAWAGRCGRIGRWG	7_2955128
7000006195550020	2955118	2955162	Rv2628	GRCGRIGRWGVHQEA	7_2955128
7000006195550040	2955358	2955402	Rv2628	DWPAAYAIGEHLSVE	7_2955392
7000006195550040	2955373	2955417	Rv2628	YAIGEHLSVEIAVAV	7_2955392
7000006195549590	4352424	4352468	Rv3874	AQAAVVRFQEAANKQ	7_4352439
7000006195549590	4352439	4352483	Rv3874	VRFQEAANKQKQELD	4.1.2.1_4352475<br>7_4352439
7000006195549590	4352454	4352498	Rv3874	AANKQKQELDEISTN	4.1.2.1_4352475
7000006195549590	4352469	4352513	Rv3874	KQELDEISTNIRQAG	4.1.2.1_4352475
7000006195549600	686965	687012	Rv0589	VAFRAGLVMEAGSKVT	4.9_686972
7000006195549610	4351141	4351194	Rv3873	PMLAAAAGWQTLSAALDA	4.1.2.1_4351160
7000006195549620	4351723	4351776	Rv3873	GPMQQLTQPLQQVTSLFS	1_4351759
7000006195549620	4351753	4351806	Rv3873	QQVTSLFSQVGGTGGGNP	1_4351759
7000006195549640	352028	352081	Rv0288	AMEDLVRAYHAMSSTHEA	6_352058
7000006195549640	352058	352111	Rv0288	AMSSTHEANTMAMMARDT	6_352058
7000006195549640	3378771	3378830	Rv3019c	YAGTLQSLGADIASEQAVLS	1_3378828
7000006195549640	3378801	3378860	Rv3019c	DIASEQAVLSSAWQGDTGIT	1_3378828
7000006195549650	3378921	3378980	Rv3019c	SMSGTHESNTMAMLARDGAE	7_3378952
7000006195549650	3378951	3378998	Rv3019c	MAMLARDGAEAAKWGG	7_3378952
7000006195549670	4352418	4352462	Rv3874	TAAQAAVVRFQEAAN	7_4352439
7000006195549670	4352430	4352474	Rv3874	AAVVRFQEAANKQKQ	7_4352439
7000006195549670	4352442	4352486	Rv3874	RFQEAANKQKQELDE	4.1.2.1_4352475
7000006195549670	4352466	4352510	Rv3874	QKQELDEISTNIRQA	4.1.2.1_4352475
7000006195549690	4352424	4352483	Rv3874	AQAAVVRFQEAANKQKQELD	4.1.2.1_4352475<br>7_4352439
7000006195549690	4352454	4352513	Rv3874	AANKQKQELDEISTNIRQAG	4.1.2.1_4352475
7000006195549730	1020433	1020477	Rv0915c	LGQNSAAIAATQAEY	5_1020452
7000006195549780	4352409	4352483	Rv3874	AAGTAAQAAVVRFQEAANKQKQELD	4.1.2.1_4352475<br>7_4352439

7000006195549780	4352454	4352528	Rv3874	AANKQKQELDEISTNIRQAGVQYSR	4.1.2.1_4352475
7000006195549790	3187480	3187554	Rv2875	GASVTVTGQGNSLKVGNADVVCGGV	4.3.1_3187535
7000006195549790	3187525	3187599	Rv2875	GNADVVCGGVSTANATVYMIDSVLM	4.3.1_3187535
7000006195549810	4357092	4357151	Rv3878	AAELAPRVVATVPQLVQLAP	4.1_4357123
7000006195549820	4266002	4266061	Rv3804c	FYSDWYQPACGKAGCQTYKW	4.4.1.2_4266036
7000006195549820	4266032	4266091	Rv3804c	GKAGCQTYKWETFLTSELPG	4.4.1.2_4266036
7000006195549850	4265981	4266040	Rv3804c	PVGGQSSFYSDWYQPACGKA	4.4.1.2_4266036
7000006195549850	4266011	4266070	Rv3804c	DWYQPACGKAGCQTYKWETF	4.4.1.2_4266036
7000006195549570	2227288	2227359	Rv1983	NGIVTAPTAVNVVLLSIPTSPFAI	7_2227339
7000006195549570	3351437	3351508	Rv2994	PSWGLVVTMFAWGYLLDHVGERMV	1_3351472
7000006195549580	4357107	4357175	Rv3878	PRVVATVPQLVQLAPHAVQMSQN	4.1_4357123

Supplementary Table 7

Sub-lineage proportions observed in the Russian dataset (n=850)[23] when using the 62-SNPs classification scheme.

Sub-lineage	Frequency
2.2.1 (Modern Beijing)	518
4.8 (T spoligotype, RD219)	71
4.2.1 (Ural)	68
4.3.3 (LAM, RD115)	57
4.1.2.1 (Haarlem, RD182)	36
4.1.2	15
4.1 (X-type)	8
3 (CAS-Delhi)	5
4.9 (H37Rv-like)	5
BOV (*M. bovis*)	4
4.3.4.1 (LAM, RD174)	3
4.3.4.2 (LAM, RD174)	3
4.4.1.1 (S type)	3
4.7	3
5 (West-Africa 1)	2
6 (West-Africa 2)	2
1.1.2 (EAI5;EAI3)	1
1.1.3 (EAI6)	1
2.2.1.1 (Beijing-RD150)	1
4.5 (RD122)	1
Probable mixed infections*	41

*The most frequent combinations of strain types were: Modern Beijing (2.2.1) with LAM (4.3.3) (n=8); Modern Beijing with Ural (4.2.1) (n=8); Modern Beijing with Haarlem (4.1.2.1) (n=4); Modern Beijing with 4.8 (n=3); and Ural with 4.8.

SUPPLEMENTARY REFERENCES

1. Zhang, H. *et al.* Genome sequencing of 161 Mycobacterium tuberculosis isolates from China identifies genes and intergenic regions associated with drug resistance. *Nature genetics* **45,** 1255–1260 (2013).

2. Firdessa, R., Berg, S. & Hailu, E. Mycobacterial lineages causing pulmonary and extrapulmonary tuberculosis, Ethiopia. *Emerging infectious …* **19,** 460–463 (2013).

3. Blouin, Y. *et al.* Significance of the Identification in the Horn of Africa of an Exceptionally Deep Branching Mycobacterium tuberculosis Clade. *PloS one* **7,** e52841 (2012).

4. Coll, F. *et al.* PolyTB: A genomic variation map for Mycobacterium tuberculosis. *Tuberculosis (Edinburgh, Scotland)* 1–9 (2014). doi:10.1016/j.tube.2014.02.005

5. Bentley, S. D. *et al.* The genome of Mycobacterium africanum West African 2 reveals a lineage-specific locus and genome erosion common to the M. tuberculosis complex. *PLoS neglected tropical diseases* **6,** e1552 (2012).

6. Joung, S., Jeon, S. & Lim, Y. Complete genome sequence of Mycobacterium bovis BCG Korea, the Korean vaccine strain for substantial production. *Genome Announcements* **1,** 87–88 (2013).

7. Orduña, P. *et al.* Genomic and proteomic analyses of Mycobacterium bovis BCG Mexico 1931 reveal a diverse immunogenic repertoire against tuberculosis infection. *BMC genomics* **12,** 493 (2011).

8. Gomes, L. H. F. *et al.* Genome sequence of Mycobacterium bovis BCG Moreau, the Brazilian vaccine strain against tuberculosis. *Journal of bacteriology* **193,** 5600–1 (2011).

9. Brosch, R. *et al.* Genome plasticity of BCG and impact on vaccine efficacy. *Proceedings of the National Academy of Sciences of the United States of America* **104,** 5596–601 (2007).

10. Seki, M. *et al.* Whole genome sequence analysis of Mycobacterium bovis bacillus Calmette-Guérin (BCG) Tokyo 172: a comparative study of BCG vaccine substrains. *Vaccine* **27,** 1710–6 (2009).

11. Roetzer, A. *et al.* Whole Genome Sequencing versus Traditional Genotyping for Investigation of a Mycobacterium tuberculosis Outbreak: A Longitudinal Molecular Epidemiological Study. *PLoS Med.* **10,** e1001387 (2013).

12. Narayanan, S. & Deshpande, U. Whole-genome sequences of four clinical isolates of Mycobacterium tuberculosis from Tamil Nadu, south India. *Genome announcements* **1,** 4430 (2013).

13. Zhang, Y. *et al.* Complete genome sequences of Mycobacterium tuberculosis strains CCDC5079 and CCDC5080, which belong to the Beijing family. *Journal of bacteriology* **193,** 5591–2 (2011).

14. Peterson, J. *et al.* Whole-genome comparison of Mycobacterium tuberculosis clinical and laboratory strains. *Journal of Bacteriology* **184,** 5479–5490 (2002).

15. Ilina, E. N. *et al.* Comparative genomic analysis of Mycobacterium tuberculosis drug resistant strains from Russia. *PloS one* **8,** e56577 (2013).

16. Rashdi, A. Al & Jadhav, B. Whole-genome sequencing and annotation of a clinical isolate of Mycobacterium tuberculosis from Mumbai, India. *Genome Announcements* **2,** 2014 (2014).

17. Gagneux, S. & Small, P. M. Global phylogeography of Mycobacterium tuberculosis and implications for tuberculosis product development. *The Lancet infectious diseases* **7,** 328–337 (2007).

18. Feuerriegel, S. *et al.* Thr202Ala in thyA is a marker for the Latin American Mediterranean lineage of the Mycobacterium tuberculosis complex rather than para-aminosalicylic acid resistance. *Antimicrobial agents and chemotherapy* **54,** 4794–8 (2010).

19. Madhavilatha, G. K. *et al.* Whole-genome sequences of two clinical isolates of Mycobacterium tuberculosis from Kerala, South India. *Journal of bacteriology* **194,** 4430 (2012).

20. Alix, E., Godreuil, S. & Blanc-Potard, A.-B. Identification of a Haarlem genotype-specific single nucleotide polymorphism in the mgtC virulence gene of Mycobacterium tuberculosis. *Journal of clinical microbiology* **44,** 2093–8 (2006).

21. Isaza, J. P. *et al.* Whole genome shotgun sequencing of one Colombian clinical isolate of Mycobacterium tuberculosis reveals DosR regulon gene deletions. *FEMS microbiology letters* **330,** 113–20 (2012).

22. Feuerriegel, S., Köser, C. U. & Niemann, S. Phylogenetic polymorphisms in antibiotic resistance genes of the Mycobacterium tuberculosis complex. *The Journal of antimicrobial chemotherapy* (2014). doi:10.1093/jac/dkt535

23. Casali, N. *et al.* Evolution and transmission of drug-resistant tuberculosis in a Russian population. *Nature genetics* **46,** 279–86 (2014).

24. Filliol, I. *et al.* Global Phylogeny of Mycobacterium tuberculosis Based on Single Nucleotide Polymorphism (SNP) Analysis : Insights into Tuberculosis Evolution , Phylogenetic Accuracy of Other DNA Fingerprinting Systems , and Recommendations for a Minimal Standard SNP Set. *J. Bacteriol.* **188,** 759–772 (2006).

25. Comas, I., Homolka, S., Niemann, S. & Gagneux, S. Genotyping of genetically monomorphic bacteria: DNA sequencing in Mycobacterium tuberculosis highlights the limitations of current methodologies. *PloS one* **4,** e7815 (2009).

26. Homolka, S. et al. High Resolution Discrimination of Clinical Mycobacterium tuberculosis Complex Strains Based on Single Nucleotide Polymorphisms. *PloS One* **7,** e39855 (2012).
